# Testing the effects of photobiomodulation on angiogenesis in a newly established CAM burn wound model

**DOI:** 10.1038/s41598-023-50165-6

**Published:** 2023-12-27

**Authors:** Bardia Firouz, Lorenz Faihs, Paul Slezak, Nassim Ghaffari Tabrizi-Wizsy, Kurt Schicho, Raimund Winter, Lars Peter Kamolz, Peter Dungel

**Affiliations:** 1 Ludwig Boltzmann Institute for Traumatology, The Research Center in Cooperation with AUVA, 1200 Vienna, Austria; 2 Division of Immunology CAM Lab, Otto Loewi Research Center, Medical University of Graz, 8036 Graz, Austria; 3 Department of Oral and Maxillofacial Surgery, Medical University of Vienna, 1090 Vienna, Austria; 4 Division of Plastic, Aesthetic and Reconstructive Surgery, Department of Surgery, Medical University of Graz, 8036 Graz, Austria; 5 Center for Regenerative Medicine and Precision Medicine-Coremed, Joanneum Research Forschungsgesellschaft mbH, 8010 Graz, Austria

**Keywords:** Biophysics, Medical research

## Abstract

Burn wounds are a common challenge for medical professionals. Current burn wound models hold several limitations, including a lack of comparability due to the heterogeneity of wounds and differences in individual wound healing. Hence, there is a need for reproducible in vivo models. In this study, we established a new burn wound model using the chorioallantoic membrane assay (CAM) as a surrogate model for animal experiments. The new experimental setup was tested by investigating the effects of the auspicious biophysical therapy, photobiomodulation (PBM), on the wound healing of an induced CAM burn wound with a metal stamp. PBM has been shown to positively influence wound healing through vascular proliferative effects and the increased secretion of chemotactic substances. The easily accessible burn wounds can be treated with various therapies. The model enables the analysis of ingrowing blood vessels (angiogenesis) and diameter and area of the wounds. The established model was used to test the effects of PBM on burn wound healing. PBM promoted angiogenesis in burn wounds on day 4 (p = 0.005). Furthermore, there was a not significant trend toward a higher number of vessels for day 6 (p = 0.065) in the irradiated group. Changes in diameter (p = 0.129) and the burn area (p = 0.131) were not significant. Our results suggest that CAM can be a suitable model for studying burn wounds. The novel experimental design enables reproducible and comparable studies on burn wound treatment.

## Introduction

Wound healing is a critical biological process in the human body that involves a complex interplay of regulated signaling pathways inducing cellular interactions^1–4^.

The CAM assay is a multifaceted alternative in vivo model. It is widely used to study angiogenesis, tumor growth, ion and immune cell transport, as well as tissue transplants^5–8^. The CAM model offers some significant advantages for scientific investigation. It consists of a highly vascularized membrane on the surface, which is easily accessible and exchanges gases and metabolites during embryonic development. It is relatively easy to use and entails low costs. Furthermore, the model can be easily adapted to the experimental prerequisites. Ex ovo incubation facilitates accessibility and imaging of the rich vascular network^5,7^.

Previous studies on wound healing in CAM have demonstrated comparability to human mechanisms to a certain extend. Significant wound healing processes were observed in the CAM, including an increase in microvessel and fibroblast density, accompanied by inflammatory infiltrate, following injury, which made the CAM become a suitable model for studying wound healing^9,29^.

However, there is a lack of research on burn wounds using this model. Burn injuries are one of the most common and devastating types of wounds, affecting millions of people worldwide^1,10^. Studying burn wound healing using the CAM could offer a valuable tool for researchers to better understand the processes involved in this complex and multifaceted healing process and identify novel therapeutic options^4^.

One of the most critical mechanisms in wound healing is angiogenesis, which ensures blood supply to the cells and ultimately contributes to tissue regeneration^5^.

Photobiomodulation (PBM) is a biophysical therapy with broad applicability in various medical fields, and its significance has increased over recent years. PBM’s angiogenic effects have been demonstrated in the CAM^1,11^. These effects are already being used for the prevention of hypertrophic scars and to improve wound healing in chronic wounds. Moreover, PBM alleviates pain and is attributed to anti-inflammatory effects, which are advantageous for wound management^1,5,9–12^.

Due to incoherent literature and the lack of standardized studies on this topic, the effectiveness of light therapy has long been questioned. In recent years, however, studies have addressed the effects of PBM and its medical applications^11,13–16^.

The mechanisms underlying the angiogenic effects are still under discussion. One of the leading hypotheses proposes an increased activity of the cytochrome-c-oxidase (CCO) enzyme by PBM, which is required to produce ATP in the mitochondrial respiratory chain. The increased ATP production has been shown to modulate a wide range of biological responses, including increased synthesis of DNA, RNA, proteins, enzymes, and other cellular components needed to enhance cell and tissue repair and regeneration^17–19^. Furthermore, light irradiation may cause the release of nitric oxide (NO), leading to enhanced tissue perfusion^20^.

Moreover, the activation of extracellular transforming-growth-factor-β (TGF-β) by reactive oxygen species (ROS), which are increased by PBM, promotes the proliferation and remodeling of wound tissue^19,21^. TGF-β plays a significant role in the chemotaxis of inflammatory cells such as macrophages and monocytes and supports the proliferation and remodeling of wound tissue by promoting migration of keratinocytes, endothelial and fibroblast cells, and matrix synthesis^19,22^.

Finding a rationale for light therapy in wound healing requires further mechanistic and translational studies. Nevertheless, due to the variability of burn wounds and the individual wound healing of the patient population in terms of age, pre-existing medical conditions, and medication, it is very challenging to perform quantitative studies with control groups on the potential effect of PBM on wound healing^11^.

Our study aimed to establish an easily accessible and reproducible burn wound model on CAM. The novel experimental setup was tested by investigating the promising effects of PBM on burn wound healing. By providing a standardized experimental protocol for this model which enables comparability and reproducibility, we aim to contribute to improvements in burn wound treatment, reducing pain and complication in wound management.

## Results

### Establishment of a burn wound model in CAM

Given the lack of literature on burn wounds on the chorioallantoic membrane, a preliminary investigation was undertaken to establish the optimal burn model on the CAM assay. Various methods of inducing a burn wound were evaluated and parameters such as wound morphology, microscopic analyzability, and injury lethality were taken into consideration.

Initially burn wounds were induced to the CAM using a surgical cautery ensuring the creation of a standardized wound area of 10mm^2^. However, we found that this method required high precision and a steady hand, making it difficult to reproduce. Furthermore, the cautery tended to stick to the surface of the CAM, leading to rupture of the membrane. Therefore, we sought an alternative method that would be more consistent and reliable.

Alternatively, we tried to induce burn wounds using a metal stamp for 3 s. We observed severe burn wounds with charring, necrotic center tissue and coagulation of adjacent blood vessels on the CAM. As a result, increased death rates of the embryos were observed, most likely primarily attributed to the early developmental stage of the CAM, which lacks the capacity to adequately compensate for blood loss resulting from vessel rupture and the extensive destruction of larger tissue areas (see SM1).

**Figure 1:**
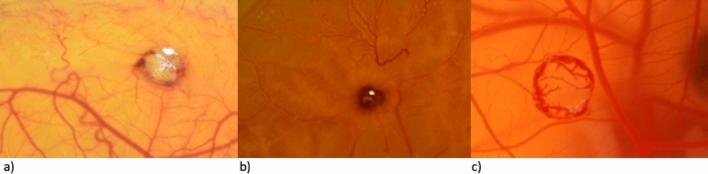
Typical examples of burn wound morphology in the CAM resulting from different inducing techniques. To establish a novel burn wound model, various methods of inducing a burn wound to the CAM were evaluated. (**a**) Surgical cautery, (**b**) metal stamp for 3 s, (**c**) metal stamp for 1 s.

Consequently, the duration of burning was reduced by decreasing contact time between the metal stamp and the CAM to 1 s. We found that the burn wounds induced for 1 s showed high survivability and smaller damage to the CAM. As a result of the intactness of the underlying tissue, as observed in second-degree human burn wounds, this group exhibited superior analyzability under the microscope. Considering these advantages, the method chosen for all our experiments was the induction of a 1 s burn wound using a metal stamp. Figure 1 presents the different methods of inducing the burn wound.

### Effects of PBM on burn wound healing

#### Diameter and area

The effects of PBM on burn wound morphology in the CAM was evaluated at given time points during the observation period. Regarding the diameter of the wounds both groups showed a significant diameter decrease with *F* (2.38, 61.77) = 403.07, *p* < 0.001 and a large effect (η^2^ = 0.94) compared to the diameter on day 0. However, no significant difference could be found between the PBM and the control group.

**Figure 2:**
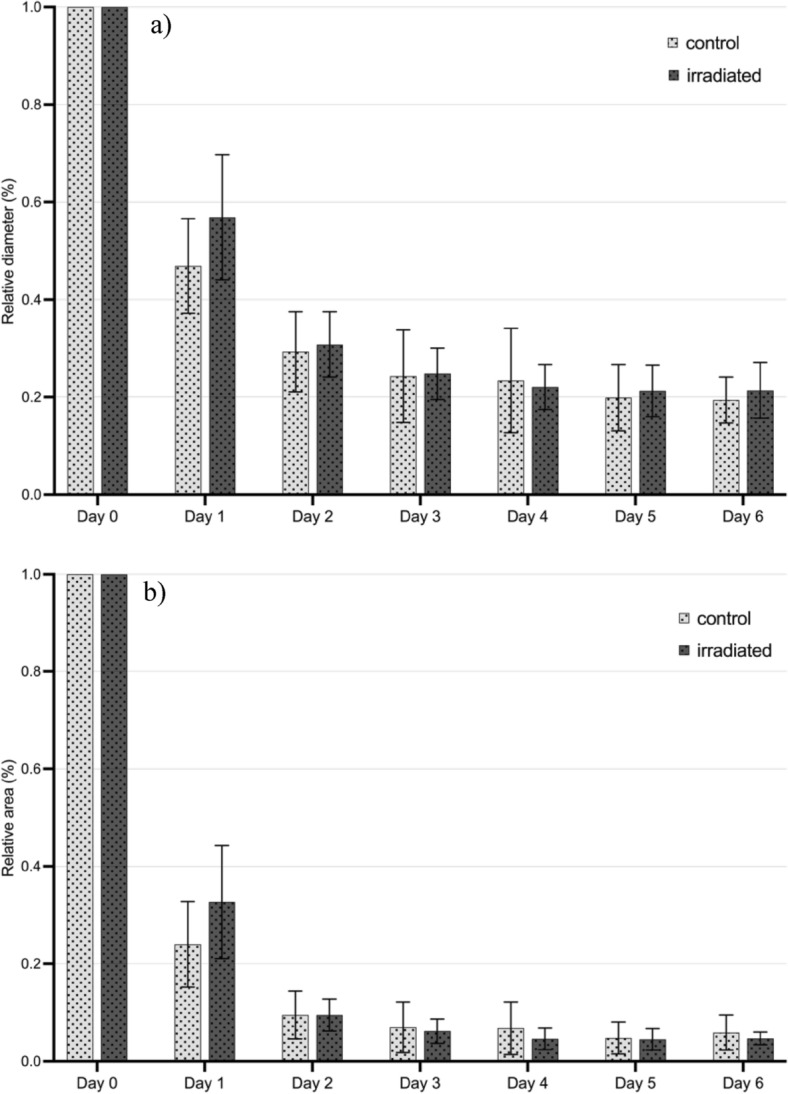
Effects of PBM treatment on burn wound morphology in the CAM. Burn wounds were placed using a metal stamp and digitally analyzed every day. Bar charts illustrate changes in the morphology of the burn wounds over time, comparing the intervention group (daily PBM treatment, dark bars) to the control group (no intervention, light grey bars). (**a**) Changes in diameter of the wound. (**b**) Changes in area of the wound. n = 28.

Examination of wound area of the burn wounds also showed a significant decrease with *F* (1.26, 32.73) = 322.36, *p* < 0.001 and a large effect (η^2^ = 0.93). Control and intervention group showed no significant difference. Results illustrating changes in the diameter and area of the wound are shown in Fig. 2.

#### Vessel sprouting

**Figure 3:**
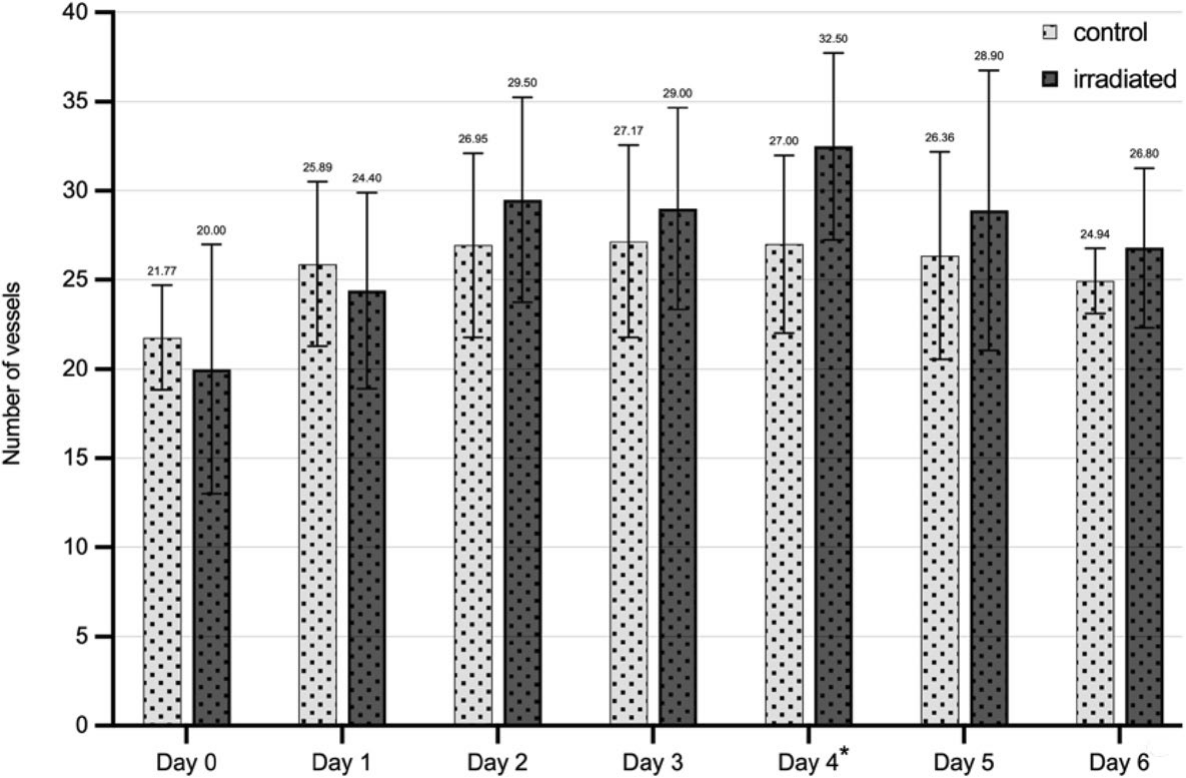
Effects of PBM treatment on vessel sprouting in the CAM burn wound model. Burn wounds were placed using a metal stamp and digitally analyzed. Bar chart illustrating the number of ingrowing vessels over time comparing the intervention group (daily PBM treatment, dark bars) to the control group (no intervention, light gray bars). n = 28, *p < 0.005, compared to control group on the same day.

Image analysis of ingrowing vessels showed a significant increase with *F* (6, 156) = 13.122, *p* < 0.001 a large effect (η^2^ = 0.34). Post-hoc comparisons were performed using one-tailed t-testing for independent samples because of the assumption that irradiation promotes angiogenesis. On day 4 of wound healing, a significantly higher number (*p* = 0.005) of ingrowing vessels could be found in wounds treated with PBM. This trend could also be observed on day 6 (*p* = 0.065). Results are shown in Fig. 3.

## Discussion

This study presents a novel experimental design to study burn wounds in CAM. With the use of this setup, we investigated the effects of PBM on burn wound healing. The CAM assay was observed to survive superficial burn wounds and can contribute to the understanding of angiogenetic effects in the human healing process.

One of the main advantages of the experimental design is its simplicity and ease of use. Unlike other animal models that require surgical procedures to create burn wounds, our model implemented a heated stamp to place burn wounds, causing a consistent and reproducible burn injury. The simplicity of the study design allows studies with large case numbers, which increases power and significance.

The CAM is an interesting alternative to animal models and provides a suitable opportunity for research in between in vitro and in vivo models^11^. Furthermore, standardized burn wounds allow direct comparison of treatment and control groups, reducing the number of chicken embryos regarding the 3R principles^23^.

The translational significance of the CAM burn wound model to the human body has yet to be investigated. The decrease in wound diameter and area and an increased number of ingrowing vessels suggest progressive healing of the burn wounds in CAM, making it a suitable model for investigating tissue regeneration in general. Nevertheless, the relevance of the reduction of the wound diameter and area must be examined histologically to investigate whether this constitutes scar formation or wound healing.

It is essential to emphasize that our CAM model allowed analysis exclusively of superficial burn wounds comparable to human 2a degree burn injuries. Our observations revealed that, during its early developmental stages, the CAM lacks the capability to survive severe burn wounds, limiting its translational potential for deeper injuries. Additionally, the absence of skin adnexa in early CAM development further constrains the applicability of the model. Yet, in the context of superficial burn wounds, we successfully identified angiogenesis. Ribatti et al. also observed processes of wound healing in the wounded CAM, such as an increase in both fibroblast density and the number of microvessels under the wound as well as inflammatory infiltrate. Morphologically, scarring or at least a reduction in the discontinuity of the membrane was observed in the majority of CAMs^9,29^. In addition, the analysis of angiogenesis in CAM seems to allow valid conclusions to be drawn for humans^19^.

While our CAM burn wound model has several advantages, there are also some limitations to consider. First, the CAM is an embryonic membrane, and as such, its inflammatory response is decreased and important steps of burn wound healing may differ from that of human tissues^24^. Second, the burn injury on the CAM is relatively small, and may not fully capture the complexity of larger burn injuries in humans. Finally, according to the 3 R-principles for minimizing animal experiments, we limited our study to 14 days, as CAMs are considered sentient beings until the 14th day of development^2,23^. Thus, delayed effects or long-term effects of the PBM treatment could not be evaluated.

We also demonstrated the effectiveness of our CAM burn wound model in evaluating the effects of a potential new treatment method for burn wounds. In both groups, a significant decrease in wound diameter and an increased number of ingrowing vessels were found. These findings can suggest healing of the burn wounds in the CAM, making it a suitable model for investigating tissue regeneration.

First, we investigated the morphology of the wounds. Our results showed a significant decrease in the diameter and area of the burn wounds in both groups, indicating the initiation of wound healing^9^. However, there was a non-significant difference in the reduction between the two cohorts.

In contrast, analysis of ingrowing blood vessels showed a significant increase in vascularity in both groups over time. A significantly higher number of ingrowing vessels for the PBM group on day four and a trended significance on day six of observation could be seen, suggesting pro-angiogenic effects in burn wound healing.

Our findings correlate with those of Winter et al., who analyzed the effects of PBM on angiogenesis in the uninjured CAM. The number of vascular branches in the daily irradiated group was significantly higher compared to the not illuminated control group^11^.

## Conclusion

In conclusion, our CAM burn wound model offers a simple and reproducible method for studying superficial burn wound healing mechanisms and evaluating potential new treatments. Its translational potential and ease of use make it a valuable addition to the existing repertoire of animal models for burn injuries. With this model, we tested the stimulating effects of Photobiomodulation on angiogenesis in the burn wounded CAM assay. We have demonstrated the survivability of our model to superficial burn wounds and its potential to analyze angiogenesis. However, we recognize the need for more optimization of this model to conclusively determine whether the reported pro-angiogenic effects of PBM can be consistently observed. Further studies are needed to validate the effectiveness of this model in assessing other treatments and to determine their optimal preclinical research. The use of imaging and histological techniques may contribute to better understand the physiological mechanisms of PBM on wound healing.

We believe that our model will pave the way for further preliminary work in this area, ultimately leading to the development of new and improved treatments for burn injuries.

## Methods

### Ex ovo cultivation

The ex-ovo CAM model was used as describes previously. Briefly, fertilized white Lohmann chicken eggs (Schropper GmbH, Gloggnitz, Austria) were incubated for three days at a temperature of 37.6 °C and constant humidity while being turned automatically in an incubator (Easy 100, J. Hemel Brutgeraete, Verl., Germany). Since the experimental conditions provide the optimal environment for the proliferation of pathogenic bacteria, all work surfaces were regularly disinfected to maintain a sterile working environment^26^. On day three of development, eggs were cracked, transferred into sterile weigh boats and covered with a petri dish, in which they were further incubated until the end of the study on day 14 of incubation.

### Burn wounds

**Figure 4:**
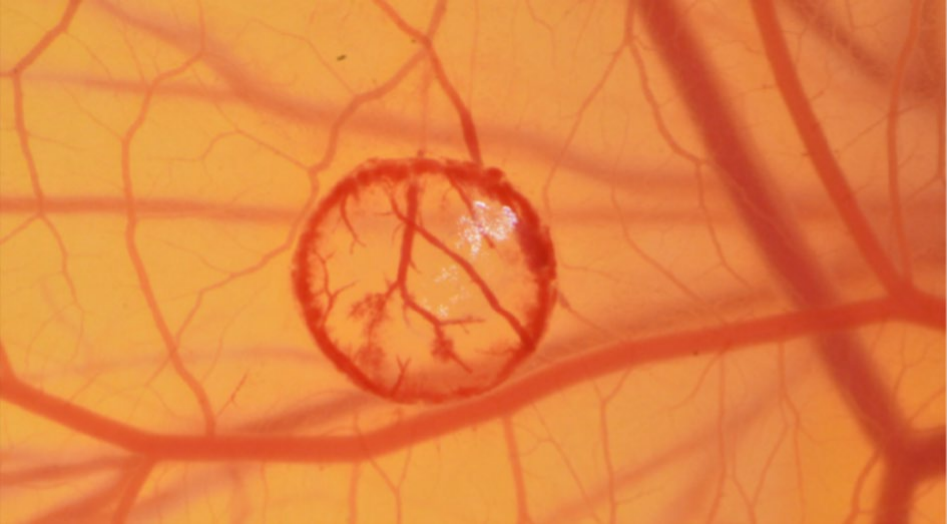
Typical microscopic image of a CAM burn wound immediately after burn wound placement. Burn wounds were placed by applying a metal stamp on the CAM for 1 s. Imaging was performed under a light microscope (Leica M651) with a SLR camera (Nikon D5600).

On the 8th day of embryonic development, a burn wound was induced on the CAM surface of all embryos using a forceps with a rounded tip, preheated to a temperature of 100 °C. A circular burn wound with a diameter of 10 mm2 was created by gently pressing the instrument on the surface of the CAM for one second. To ensure experiment reproducibility, precise temperature measurements were obtained using an infrared thermometer. The placement of the burn wounds was carefully chosen in areas of interest between major blood vessels, to visualize the ingrowth of capillaries while minimizing the risk of lethal injuries. Following the induction of the burn wounds, the CAMs were numbered and randomly assigned to either a control or intervention group using specialized randomization software. Figure 4 shows the surface of the chorioallantoic membrane right after placing a burn wound.

### Photobiomodulation

**Figure 5:**
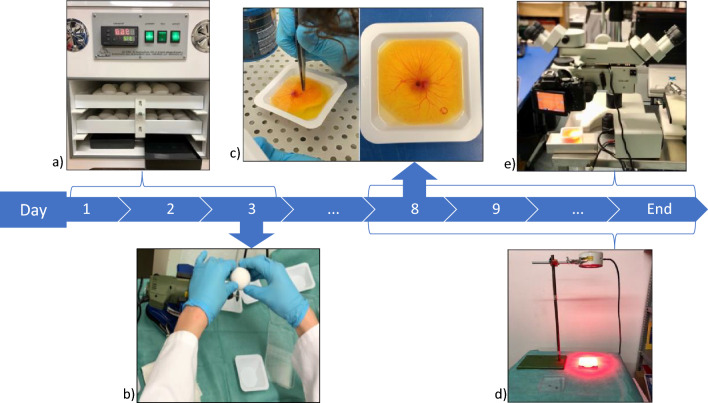
Experimental setup: (**a**) start of incubation. (**b**) Cracking the eggs into sterile weight boats. (**c**) Placing burn wounds on the CAM. (**d**) Applying PBM to the CAM. (**e**) Imaging.

From the day of wounding, the embryos of the intervention group were irradiated daily by PBM. Light exposure was applied with the repuls® 7 radiator (Repuls Lichtmedizintechnik GmbH, Austria)^27^. This device emits pulsed LED light of 635 nm (red) at a pulse rate of 50% and a repetition frequency of 2.5 Hz. The duration of irradiation was set at 12 min. The distance to the CAM surface was set to 15 cm according to the manufacturer’s instructions, ensuring irradiation of the entire membrane (see SM2). Figure 5 presents an overview of the experimental setup.

### Image analysis

CAMs of both groups were imaged from the day wounding for six consecutive days. Photo documentation was performed using a light microscope (model: Leica M651, Leica Microsystems, Wetzlar, Germany), to which a commercial SLR camera (model: Nikon D5600, Nikon Corp, Tokyo, Japan) was mounted. Afterwards, all images were analyzed using the software ImageJ (version 1.53a) for macOS concerning the previously defined parameters of diameter and area of the wound, as well as vessel sprouting.

**Figure 6:**
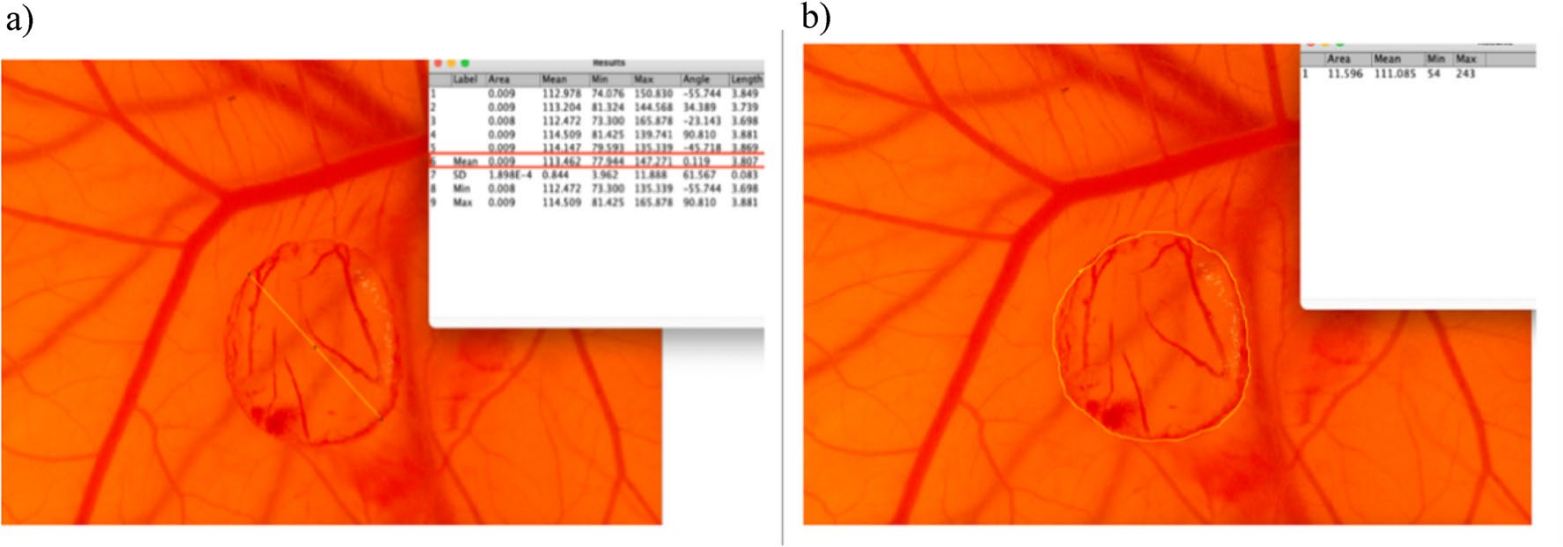
Example of CAM burn wound morphology as seen under the microscope. (**a**) Several lines were placed across the burn wound to evaluate the mean diameter (yellow line) using ImageJ. (**b**) The “freehand selection” tool in ImageJ was used to completely mark the outer edge of the burn wound and evaluate its area.

To assess the diameter of the wound, several lines were drawn between two opposing points of the burn wound using the "Line tool" in ImageJ. The mean value was then used for statistical analysis. To measure the area of the wounds as accurately as possible, the area was encircled using the "freehand-selection" function in ImageJ. Diameter and area calculation are shown in Fig. 6.

To analyze vessel sprouting, the burn was assumed to be approximately circular in this study. First, a circle with a distance of 1 mm to the outer edge of the wound was drawn. We maintained a consistent 1 mm distance from the outer edge of the burn wound for vessel ingrowth analysis, resulting in a proportionally shrinking region of interest during the wound healing process.

**Figure 7:**
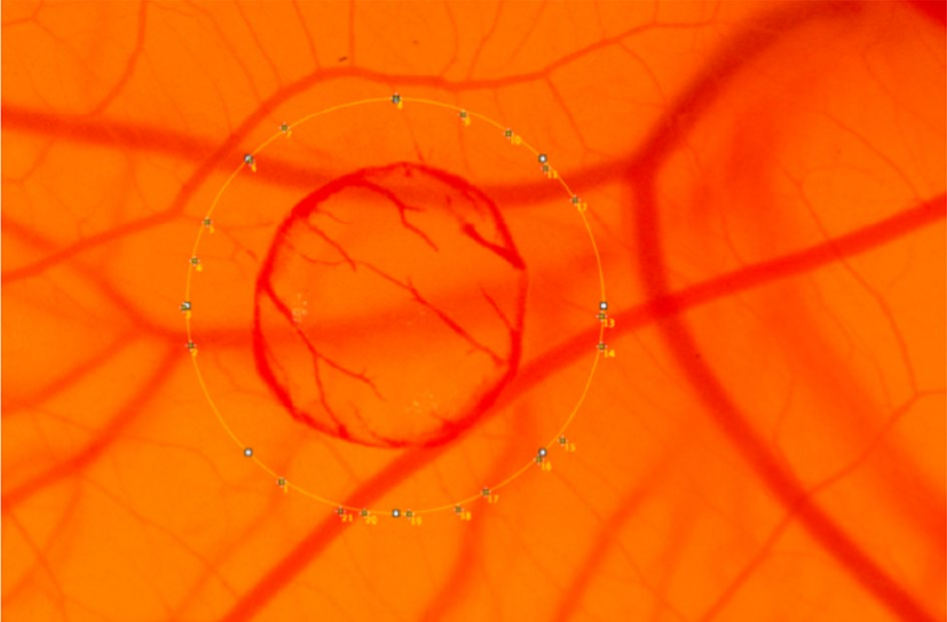
Typical example of the analysis of ingrowing vessels to the burn wound. A circle of interest with a defined diameter was placed on the Image using ImageJ. Vessels cutting the circle were marked (white dots) and manually counted as ingrowing vessels.

Afterwards, intersecting vessels were marked and manually counted. Figure 7 shows the manual count of ingrowing vessels.

### Statistical analysis

Statistical analysis was performed using two-way mixed ANOVA models comparing changes in study-relevant parameters considering the two conditions. For all statistical tests, a p-value < 0.05 was considered statistically significant.

In multiple testing, Bonferroni adjustment according to α* = α/k was used to avoid α-risk Standardized effect measures according to Cohen’s classification were also used to interpret the practical relevance of results^28^. All statistical analyses were performed with SPSS for macOS, Version 28 (IBM, Armonk, NY, USA).

### Supplementary Information

Supplementary Information.

## Data Availability

The datasets used and/or analyzed during the current study available from the corresponding author on reasonable request.
