# Supplementary material for: Testing the effects of photobiomodulation on angiogenesis in a newly established CAM burn wound model
Source: Sci Rep. 2023 Dec 27;13:22985. doi: 10.1038/s41598-023-50165-6 (PMC10752885; doi:10.1038/s41598-023-50165-6)
Supplement: Supplementary file 1 — Supplementary Information. [file 41598_2023_50165_MOESM1_ESM.pdf]

Testing the effects of photobiomodulation on angiogenesis in a newly established CAM burn wound model

Supplementary material:

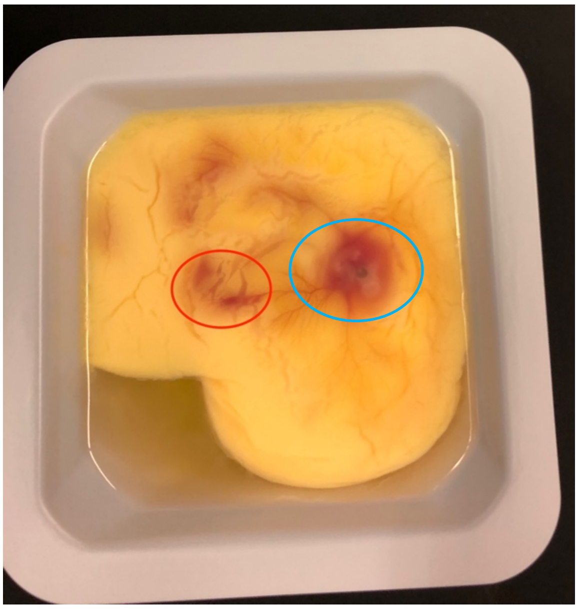

Supplement 1: A deceased CAM embryo is shown one day after inducing a severe burn wound. The red circle highlights the site of burn wound induction, indicating significant blood loss that led to the demise of the CAM. After demise, there is a subsequent atrophy observed in the rich vascular network and the embryo (blue circle).

Model	repuls® 7
Number of LEDs	7
Wavelenght	620-640 nm
Light intensity	175 mW
Total light output	4100 mW
Pulse frequency	2.5 Hz
Treatment-time	6-12 min
Medicinal product	IIb

Supplement 2: Technical specifications of the Repuls 7 irradiator.
